# Fatty Acid and Phospholipid Syntheses Are Prerequisites for the Cell Cycle of *Symbiodinium* and Their Endosymbiosis within Sea Anemones

**DOI:** 10.1371/journal.pone.0072486

**Published:** 2013-08-29

**Authors:** Li-Hsueh Wang, Hsieh-He Lee, Lee-Shing Fang, Anderson B. Mayfield, Chii-Shiarng Chen

**Affiliations:** 1 National Museum of Marine Biology and Aquarium, Checheng, Pingtung, Taiwan; 2 Graduate Institute of Marine Biotechnology, National Dong Hwa University, Hualien, Taiwan; 3 Department of Sport, Health and Leisure Studies, Cheng Shiu University, Kaohsiung, Taiwan; 4 Living Oceans Foundation, Landover, Maryland, United States of America; 5 Department of Marine Biotechnology and Resources, National Sun Yat-Sen University, Kaohsiung, Taiwan; Centre National de la Recherche Scientifique, Aix-Marseille Université, France

## Abstract

Lipids are a source of metabolic energy, as well as essential components of cellular membranes. Although they have been shown to be key players in the regulation of cell proliferation in various eukaryotes, including microalgae, their role in the cell cycle of cnidarian-dinoflagellate (genus *Symbiodinium*) endosymbioses remains to be elucidated. The present study examined the effects of a lipid synthesis inhibitor, cerulenin, on the cell cycle of both cultured *Symbiodinium* (clade B) and those engaged in an endosymbiotic association with the sea anemone *Aiptasia pulchella*. In the former, cerulenin exposure was found to inhibit free fatty acid (FFA) synthesis, as it does in other organisms. Additionally, while it also significantly inhibited the synthesis of phosphatidylethanolamine (PE), it did not affect the production of sterol ester (SE) or phosphatidylcholine (PC). Interestingly, cerulenin also significantly retarded cell division by arresting the cell cycles at the G_0_/G_1_ phase. Cerulenin-treated *Symbiodinium* were found to be taken up by anemone hosts at a significantly depressed quantity in comparison with control *Symbiodinium*. Furthermore, the uptake of cerulenin-treated *Symbiodinium* in host tentacles occurred much more slowly than in untreated controls. These results indicate that FFA and PE may play critical roles in the recognition, proliferation, and ultimately the success of endosymbiosis with anemones.

## Introduction

Lipids are important components of all living organisms, as they are a source of metabolic energy and serve as essential components of cellular membranes. They are also involved in processes such as cell proliferation, cell differentiation, and organ morphogenesis, which are all intimately associated with the progression of the cell cycle [1]. For instance, the concentration of phospholipids in the photosynthetic bacterium *Rhodopseudomonas sphaeroids* doubles before cell division [2]. Polar glycerolipids (glycolipid, phosolipid, and ether lipid) are synthesized sequentially during the cell cycle of *Chlamydomonas reinhardtii* [3]. In the heterotropic dinoflagellate, *Crypthecodinium cohnii*, cells exhibit a stepwise increase in polar lipids and a continuous increase in neutral lipids over the course of the cell cycle [4]. The same study showed that inhibiting lipid synthesis caused cell cycle arrest at the early G_1_ phase, not the G_2_/M phase, demonstrating the essential role of lipid synthesis in regulating cell cycle progression.

The Cnidaria-*Symbiodinium* association is an endosymbiosis in which the dinoflagellate symbionts reside within the anthozoan host's gastrodermal cells and contribute to the latter's nutrition by translocating photosynthetically fixed carbon and other metabolites into the host cytoplasm [5]. Among the photosynthates produced by the symbionts, lipids and their roles in regulating the association with hosts are being subjects for intensive studies [6]–[11]. Whether all lipid synthates of symbionts would be transferred to the host remains to be determined, as a recent study of lipidomic examination has shown that the free fatty acids of symbionts did not translocate to hosts [10]. Nonetheless, elevated temperature-induced bleaching events in which *Symbiodinium* photosynthesis was impaired, led to reduced productions of neutral lipids such as triacylglycerols (TAGs) and wax ester (WE) in both *Porites compressa* and *Montipora verrucosa* [7]. Furthermore, the formation of lipid bodies (LBs) in the gastrodermal cells of *Euphyllia glabrescens* has been shown to depend upon the presence of symbionts [12]. Although the cellular mechanism of LB formation remains to be elucidated, concentrations of three major lipids of LBs (TAGs, WE and sterol) are regulated by the diel cycle and photosynthesis [13]. The depletion or alter of these lipid energy reserves also increase susceptibility to diseases and mortality, possibly through an increase in microbes in the damaged tissue [14], [15]. It is thus conceivable that the role of lipid synthesis of symbionts during the symbiosis could be pivotal and remains to be elucidated.

It has been shown that external 12 hr light/12 hr dark (12L/12D) stimulation drives the cell cycle propagation of cultured, clade B *Symbiodinium* [16]. The 12L/12D treatment entrained a single cell cycle from the G_1_ to the S phase, and then to the G_2_/M phase. Blue light (450±10 nm) alone mimics regular white light, while red (660±10 nm) and infrared (735±10 nm) lights have little or no effect on the cell cycle [16]. *In hospite*, the picture becomes more complicated, as it has been suggested that *Symbiodinium* cell proliferation may be under the regulation of host cells. For example, the frequency of *Symbiodinium* division in fed anemones is two times higher than in starved specimens [17]. Moreover, *Symbiodinium* densities vary little in healthy symbioses [18], [19], possibly because newly generated *Symbiodinium* cells are released into the host gastrovascular cavity [20].

However, lipids may also play a role in regulation of the cell cycle in stable endosymbiotic associations. In order to gain more insight into this notion, cultured *Symbiodinium* (clade B) were treated with 2,3-epoxy-4-oxo-7,10-dodecadienamide (cerulenin), a fungal metabolite [21], [22] that specifically inhibits three types of fatty acid synthases in various eukaryotes [23]. It was hypothesized that treatment with cerulenin might alter the lipid synthesis and cell cycle progression of cultured *Symbiodinium*. Our current study also focused on the ability of cerulenin-treated *Symbiodinium* to infect sea anemones, aiming to elucidate the role of lipid synthesis in the establishment of anemone-dinoflagellate endosymbiosis.

## Materials and Methods

### *Symbiodinium* culture and identification

*Symbiodinium* cells isolated from *A. pulchella* were cultured at 25°C at 40 μmol m^−2^s^−1^ over a 12 h light/12 h dark (12L/12D) cycle, and f/2 medium (Sigma, USA) containing antibiotics (10 μg ml^−1^ streptomycin and 10 units ml^−1^ penicillin; Invitrogen, USA) was replenished every 5 days. Restriction fragment length polymorphism (RFLP) analysis of the *Symbiodinium* 18S rDNA was used to identify the clade of the cultured *Symbiodinium* following a published procedure [24]. The RFLP genotype pattern 885/505 bp for *Taq* I (New England Biolabs, USA) and 765/500 bp for *Sau3A* I (New England Biolabs, USA) confirmed that the cultured *Symbiodinium* were of clade B.

### Flow cytometry analyses of cell cycle progression

**Figure 1:**
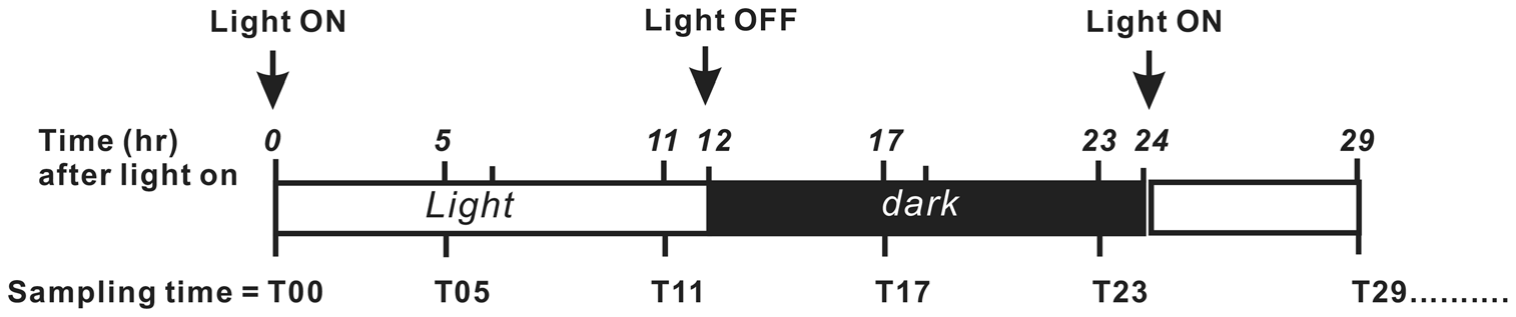
Sampling time for *Symbiodinium* sp. during a light–dark photoperiod.

The cell cycle progression was determined by flow cytometry according to a published procedure [16]. Briefly, three cultures maintained at the exponential growth stage (5×10^4^ cells ml^−1^) were sampled for analyses. 10^6^ cells were collected at the times indicated in Fig. 1. Cells were harvested by centrifugation at 68×g for 5 min and fixed in ice-cold 70% ethanol for 1 h at 18 μmol m^−2^s^−1^ to extract the chlorophyll. Cells were then incubated in phosphate-buffered saline (PBS) containing Triton-X 100 (0.1%, Pharmacia Biotech, Sweden), RNase (10 μg ml^−1^, Sigma, USA), and propidium iodide (PI; 30 μg ml^−1^, Invitrogen, USA) at 4°C overnight in the dark. The DNA content per cell calculated from the DNA–PI complex fluorescence under 488/610 nm (Excitation/Emission) with an EPICS ALTRATM flow cytometer (Beckman Coulter, Inc., USA). Histograms of relative DNA content were analyzed using MultiCycle AV for Windows V5.0 (Phoenix Flow Systems, CA) in order to quantify the percentage of cells at each stage: G_1_, S, and G_2_/M.

### Cerulenin treatments

Cerulenin (Sigma, USA), a fatty acid synthesis inhibitor, was used to modulate the lipid synthesis in cultured *Symbiodinium*, in order to examine the role of lipid synthesis in cell cycle progression. To determine the optimal concentration required to elicit a biological response, cerulenin (in DMSO) was added to *Symbiodinium* cultures to final concentrations of 0, 10^−7^, 10^−6^, or 10^−5^ M (final DMSO concentration: 0.02%) at T00 (time zero before the light on, see Fig. 1). The effect of cerulenin on cell cycle propagation of treated *Symbiodinium* during a 12L/12D photoperiod was then analyzed by flow cytometry as described in the preceding section, with three biological replicates sampled at T00, as well as after 5 (T05), 11, (T11), 17 (T17), 23 (T23), and 29 (T29) h. Lipid analyses were assessed in a separate aliquot from the same cultures of each of the four concentrations of cerulenin at T00, T05, T11, and T17.

### Analysis of lipids and starch

Cells (10^7^) from each treatment were collected at specific times after centrifugation (68×g for 5 min), and the pellets were stored at −80°C. Before assays, cells were re-suspended in PBS and homogenized using a ball mill (Retsch MM301, USA) with glass beads (425–600 μm, Sigma, USA). Each cell homogenate was quantified for total protein using Pierce BCA Protein Assay Kit (Thermo Scientific, USA) and then cell homogenate containing exactly twenty-five microgram total protein was used to assay the lipid profiles and starch contents.

Lipids were extracted by methyl tert-butyl ether (MTBE; Merck, Germany) according to Matyash et al. [25]. Methanol (1.5 ml) was added to a 200 μl sample aliquot, and the tube was vortexed. Then, 5 ml of MTBE was added and the mixture was incubated for 1 h at room temperature in a shaker. The phase separation was induced by adding 1.25 ml of deionized water. Upon 10 min of incubation at room temperature, the sample was then centrifuged at 1,000×g for 10 min. The upper organic phase was collected; and the lower phase was re-extracted with 2 ml of MTBE/methanol/water (10∶3∶2.5, v/v/v) to collect the upper organic phase. The collections of organic phases containing extracted lipids were combined and then dried by Rotary Evaporator (Panchum Scientific Corp, Taiwan).

The extracted lipids were analyzed by a high-performance liquid chromatography equipped with evaporative light scattering detector (HPLC-ELSD) [26]. A Hitachi Model L7100 HPLC pump, equipped with an auto-sampler (L7200, Hitachi, Japan), was used with a Sedex 80 evaporative light-scattering detector (Sedere, France). The drift-tube temperature was maintained at 55°C, and the flow-rate of the nebulizer gas (nitrogen) was 2.5 kg/cm^2^. The detector response was quantified by electronic integration. Solvents were de-aerated with nitrogen gas. A column of YMC-PVA-SIL (100×3 mm i.d.; 5 mm particles) was obtained from Hichrom Ltd. (Reading, UK). This experiment required a ternary gradient elution scheme consisting of isohexane (Merck, Germany) (solvent A), propan-2-ol (Merck, Germany): acetonitrile (Merck, Germany): butan-2-one (butan-2-one or 2-butanone) (Merck, Germany) (7∶2∶1, v/v/v; solvent B), and propan-2-ol:acetonitrile: butan-2-one: methanol (Merck, Germany): water: N-ethylmorpholine (Sigma, USA): acetic acid (Merck, Germany) (56∶14∶7.2∶14∶8.4∶0.42∶0.15 v/v; solvent C), with the gradient elution program described as in Table 1.

**Table 1:** Gradient elution program for HPLC separation of lipids.

Time (min)				Flow rate
	Solvents			(ml/min)
	A (%)	B (%)	C (%)	
0	100	0	0	1.0
4	100	0	0	1.0
5	85	15	0	1.0
10	80	20	0	1.0
12	75	25	0	1.0
15	50	50	0	1.0
18	30	50	20	1.0
20	30	40	30	1.0
25	25	30	45	1.0
30	30	70	0	1.0
40	100	0	0	1.0

solvent A: isohexane.

solvent B: propan-2-ol: acetonitrile:butan-2-one (butan-2-one or 2-butanone) (7∶2∶1, v/v/v).

solvent C: propan-2-ol:acetonitrile: butan-2-one: methanol:water:*N*-ethylmorpholine: acetic acid  = 56∶14∶7.2∶14∶8.4∶0.42∶0.15 v/v/v/v/v/v/v.

**Figure 2:**
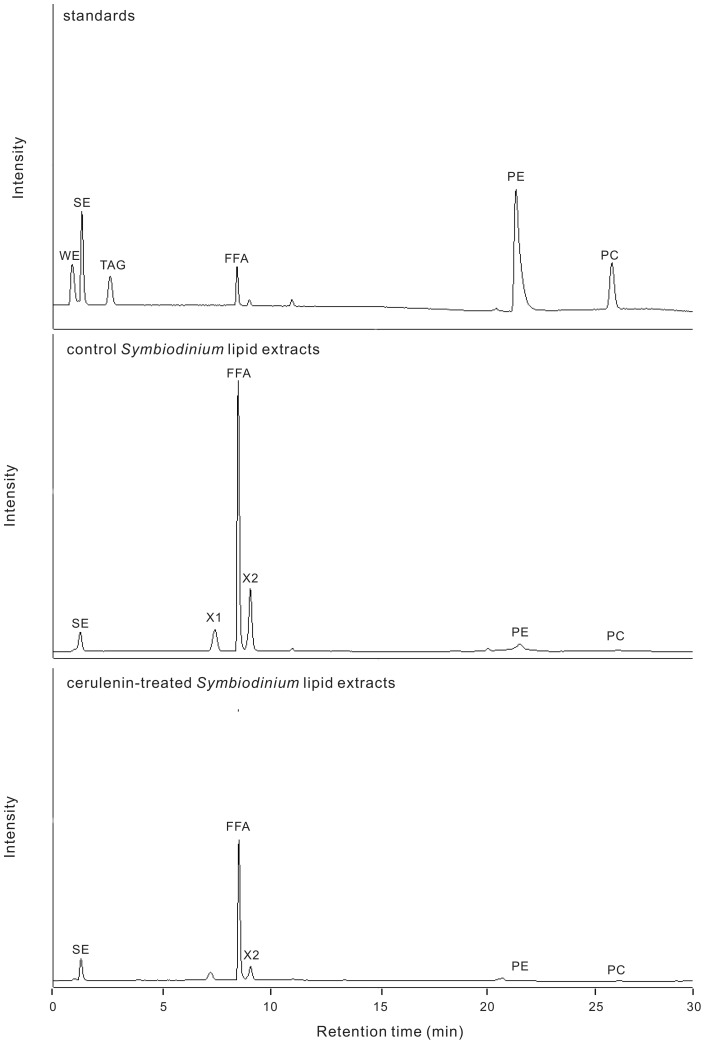
HPLC profiles of standard lipids and extracted lipids from control and cerulenin-treated *Symbiodinium* at T11. WE, wax ester; SE, sterol ester; TAG, triacylglyceride; FFA, free fatty acid; PE, phophatidylethanolamine; PC, phosphatidylcholine; X1, unknown peak 1; X2, unknown peak 2.

As shown in the upper panel of Fig. 2, six major lipid standards (from Sigma, USA and Lipid Products, England) could be clearly separated and analyzed by the current HPLC analysis, including wax ester (WE: arachidyl dodecanoate; RT  = 0.9 min), sterol ester (SE: cholosteryl oleate; RT  = 1.4 min), triacylglycerol (TAG: trilaurin; RT  = 2.9 min), free fatty acid (FFA: linoleic acid; RT  = 8.9 min), phophatidylethanolamine (PE; RT  = 22.1 min), and phosphatidylcholine (PC: lecithin; RT  = 26.8 min). In comparison with the lipid standards, HPLC analyses of lipid extracts from both control (0.02% DMSO, middle panel of Fig. 2) and cerulenin-treated (the lower panel of Fig. 2) *Symbiodinium* were lack of significant amount of WE and TAG. On the other hand, they contained two unidentified lipid species (X1 and X2 with RT at 7.8 and 9.3 min, respectively). It was confirmed that they are not wax esters, triacylglycerides, monogalactosyldiacylglycerols, digalactosyldiacylglycerols, nor phosphatidylserines, and thus were not analyzed further in the present study. As a consequence, the integrated areas in the HPLC profile of four different concentrations of lipid standards (i.e. SE, FFA, PE and PC) were used to create the linear regression equation (*Y* = *aX*+*b*, *Y* = lipid concentration and *X*  =  integrated area). The lipid concentration of *Symbiodinium* extracts was then calculated by interpolating into the lipid standard equation. Final lipid concentrations were then acquired by the normalization based on protein concentration of the extracted sample.

The starch content of each sample was assayed using a Starch Assay Kit (Sigma, USA), and normalized to the total protein concentration.

### Preparation of bleached *Aiptasia pulchella* specimens

Endosymbiotic sea anemones, *Aiptasia pulchella*, were cultured in a circulating seawater tank with temperature maintained at 25°C. Small endosymbiotic anemones (oral disc diameters ∼1 to 2 mm) were treated with cold seawater (4°C) for 5 h. Afterwards, sea anemones were immediately returned to 25°C seawater, resulting in the release of *Symbiodinium* from these samples. Bleached sea anemones were fed with *Artemia sp.* every five days and maintained in the dark for at least 6 months. They were transferred to new containers and reared in filtered (0.22 μm) seawater to prevent *Symbiodinium* re-infection. The small (oral disc diameter ∼1 mm), bleached sea anemones were first examined using a fluorescence stereomicroscope (Discovery V8, Zeiss, Germany) and subsequently cultured at 40 μmol m^−2^s^−1^) over a 12L/12D cycle for 10 d before being re-examined with a fluorescence stereomicroscope. To ensure that there were no residual *Symbiodinium* living within tissues, only completely bleached anemones were used for the infection experiment.

### Infection of Aiptasia pulchella with Symbiodinium

To prepare *Symbiodinium* for reinfection of bleached *A. pulchella*, cultured *Symbiodinium* at T00 were treate ml d with or without 10^−6^ M cerulenin for 11 h under light irradiations. These cerulenin-treated *Symbiodinium* were then harvested at T11 (68×g for 5 min), and re-suspended in seawater for infection experiments.

After being washed twice with filtered seawater (FSW), the bleached sea anemones were incubated with 10^4 −1^ of the control or cerulenin-treated *Symbiodinium* cells for 1 h. The infected sea anemones were then washed twice with FSW, moved to a new container with FSW, and cultured in across a 12L/12D cycle. The numbers of *Symbiodinium* inside sea anemones (The number of sea anemones investigated at each time points is 31, N = 31) were then examined under an epifluorescence microscope (Axiovision, Zeiss, Germany) after 6, 24, 72, and 120 h of culture.

In order to identify the intracellular distribution of *Symbiodinium* in host tissue, some anemones were fixed in 3.6% paraformaldehyde (two hours at room temperature) for histological examination. Anemones were then washed with 200 mM phosphate buffer twice, dehydrated by a series of increasing ethanol concentrations (50%, 75%, 95%, 95%, 100% and 100%, for 30 minutes each). They were infiltrated with JB-4 catalyzed solution A (Electron Microscopy Sciences, USA) for overnight at 4°C, followed by the embedding with embedding medium (2 ml JB-4 catalyzed solution A plus with JB-4 solution B). After the sample is solidified, a microtome (Leica, Germany) was used to section tissue at 5 μm thickness. Each section was then stained with hematoxylin (Merck, Germany) and trichrome (Sigma, USA) to examine the distribution of ingested *Symbiodinium* by the microscope (Axiovision, Zeiss, Germany).

### Statistical analysis

In order to determine the statistical significance of the treatments, student's t-tests or one-way analysis of variance (ANOVA) followed by Duncan's multiple-range procedure (p<0.05) were used. Non-parametric Mann-Whitney Rank Sum Tests were performed on non-normal data.

## Results

### Inhibitory effects of cerulenin on the *Symbiodinium* cell cycle

**Figure 3:**
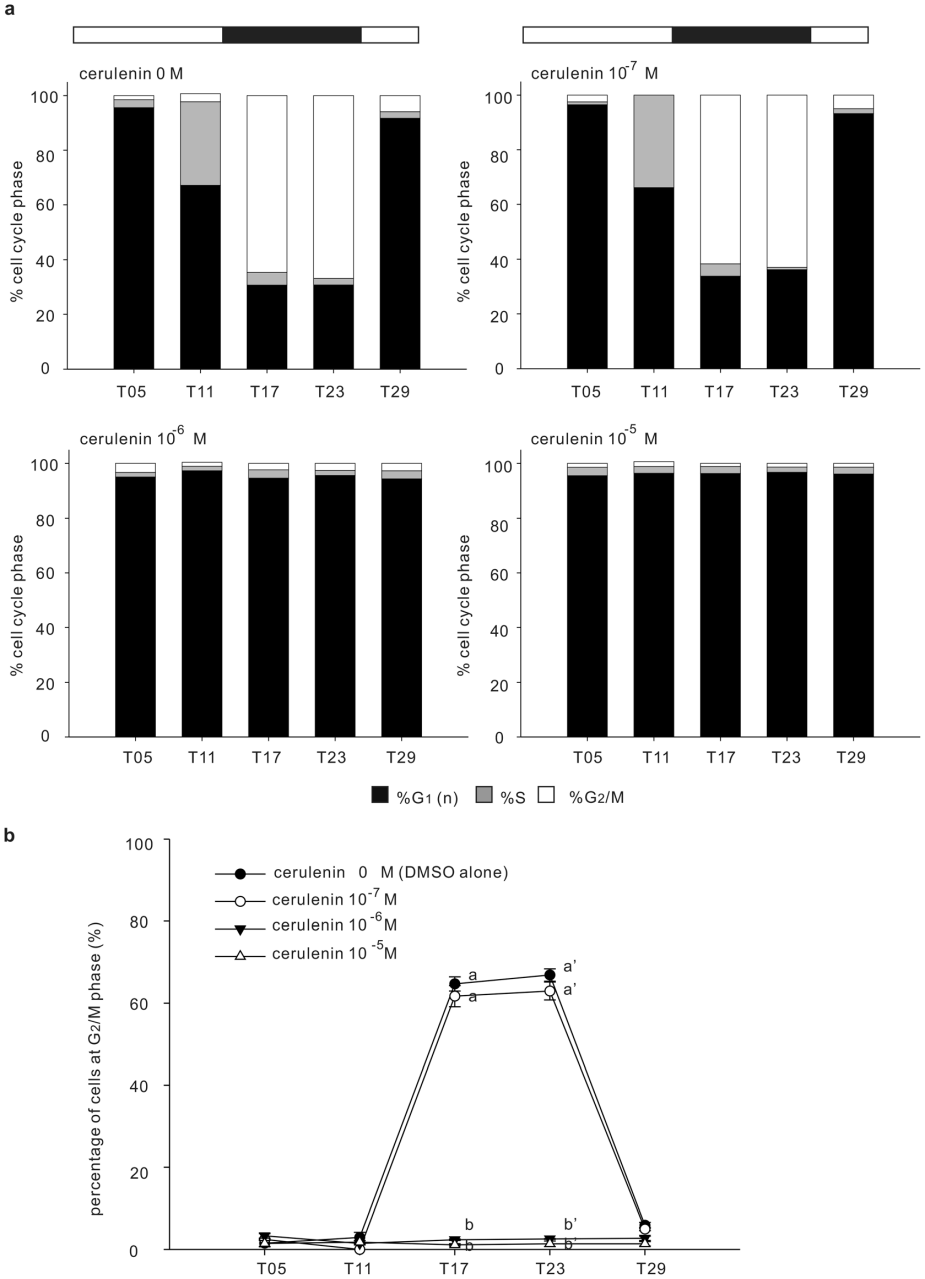
Dose-dependent response of cerulenin on the *Symbiodinium* cell cycle. Cell cycle analyses of *Symbiodinium* sp. were performed across a 12L/12D treatment in the absence (0 M) or presence of cerulenin (10^−7^ M, 10^−6^ M, and 10^−5^ M). (panel a). The percentage of cells at each stage of the cell cycle. (panel b). Values were expressed as mean ± SEM. Letters (a–b and a' –b') denote the statistical significance of different cerulenin concentrations according to one-way ANOVA followed by Duncan's multiple-range procedure (*p*<0.05).

The cell cycle propagation of control *Symbiodinium* (clade B) from the growing/DNA synthesis stage (i.e., G_1_-S-G_2_/M) to cytokinesis (i.e., G_2_/M-G_1_) was entrained by the 12L/12D cycle (Fig. 3a). Cells progressed from G_1_ (T05) to S (T11), and then to the G_2_/M phase during the first 12 h of light stimulation. During darkness (T17), DNA synthesis was greatly decreased, and more cells entered the G_2_/M phase followed by cytokinesis to generate G_1_ cells for the next cycle (T29 of Fig. 3b). Approximately 50–60% of the G_1_ cells progressed through the entire cell cycle for each 12L/12D cycle.

To determine the optimal concentration of cerulenin required to interfere with the cell cycle progression, cerulenin was added at T00 in three different concentrations: 10^−7^, 10^−6^, or 10^−5^ M (Fig. 3a). At both 10^−6^ and 10^−5^ M, cerulenin effectively arrested the cell cycle at the G_1_ phase and greatly decreased the progression toward G_2_/M. On the other hand, cerulenin was not able to alter the cell cycle progression at 10^−7^ M (Fig. 3b; F = 16.374, *p*<0.001 at T11; F = 351.483, *p*<0.001 at T17; F = 358.025, *p*<0.001 at T23). Consequently, cerulenin at a 10^−6^ M concentration was used in the following experiments.

**Figure 4:**
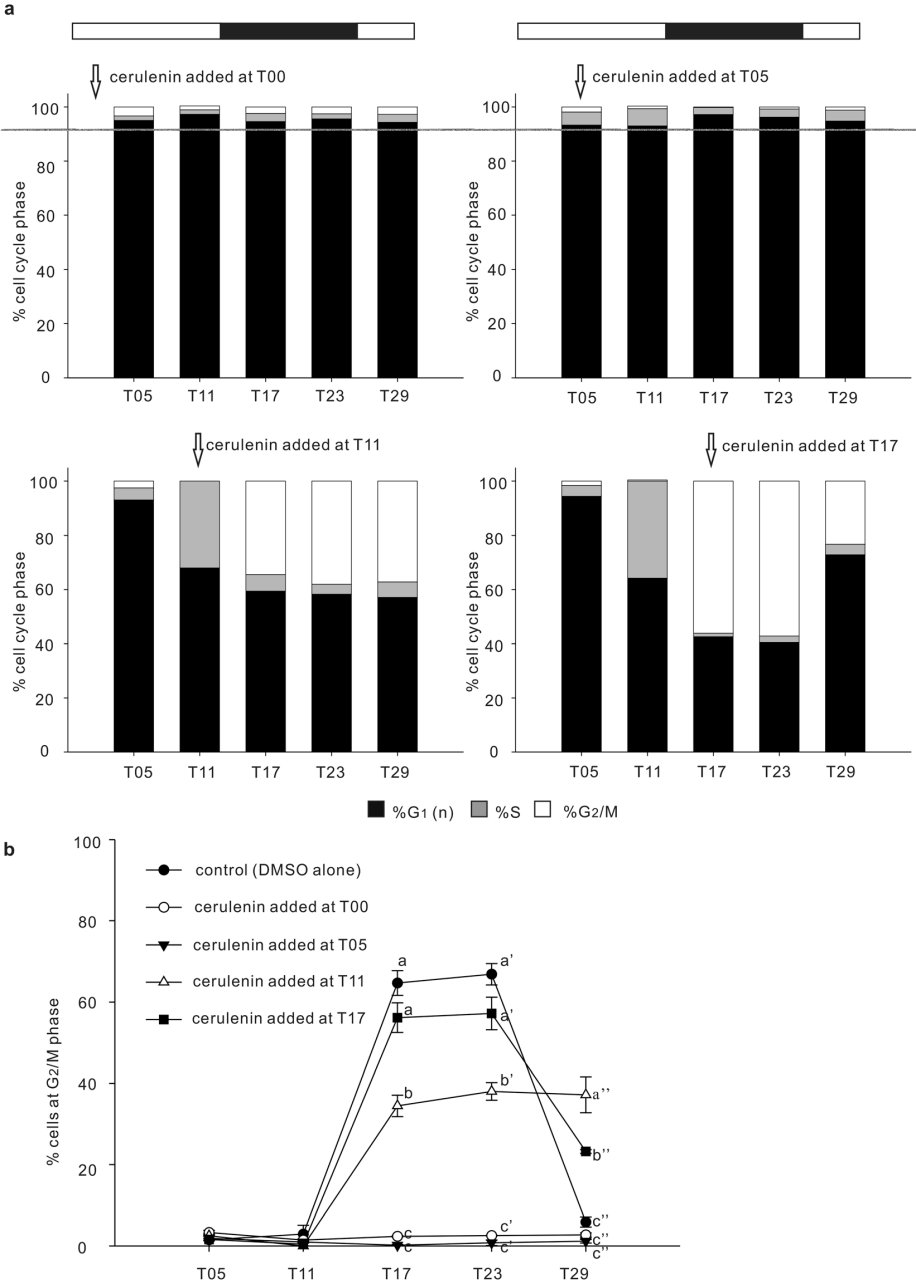
Effect of cerulenin on different stages of the *Symbiodinium* cell cycle. Cell cycle analyses of *Symbiodinium* sp. were performed across a 12L/12D photoperiod with cerulenin (10^−6^ M) added at different times (panel a). The percentage of cells at each stage of the cell cycle are shown with bar graphs. (panel b). The percentage of cells at the G_2_/M phase at the five treatments across five sampling times. Values were expressed as mean ± SEM. Letters (a–c and a'–c') denote the statistical significance of different time points according to one-way ANOVA followed by Duncan's multiple-range procedure (*p*<0.05). F = 1.889, *p* = 0.187 at T05; F = 3.444, *p* = 0.051 at T11; F = 182.065, *p*<0.001 at T17; F = 192.214, *p*<0.001 at T23; F = 57.748, *p*<0.001 at T29).

To further examine the effect of cerulenin on specific cell cycle phases, *Symbiodinium* were incubated with 10^−6^ M cerulenin at different times (T00, T05, T11, or T17) during the light-dark cycle (Fig. 4). As shown in Fig. 4a, when cerulenin was added at T00 or T05 when most cells were in the G_1_ phase, treated cells were unable to progress throughout the remainder of their cell cycle over the duration of the experiment and remained at G_1_ phase. Furthermore, when added at T11, at which point the majority of cells were either at the G_1_ or S phase (Fig. 4a), cerulenin treatment significantly decreased the percentage of cells transitioned from S to G_2_/M between T17 and T23 (see Fig. 4b; F = 182.065, *p*<0.001 at T17; F = 192.214, *p*<0.001 at T23 in comparison to control), which further resulted in a delay of mitotic division at T29 (Fig. 4b, F = 57.748, *p*<0.001). This indicates that cerulenin was also able to decrease the transition from S to G_2_/M. Cerulenin was also able to inhibit the mitotic division of G_2_/M cells when it was added at T17 (Fig. 4a–b).

### Effect of cerulenin on lipid contents of cells at different phases of the cell cycle

**Figure 5:**
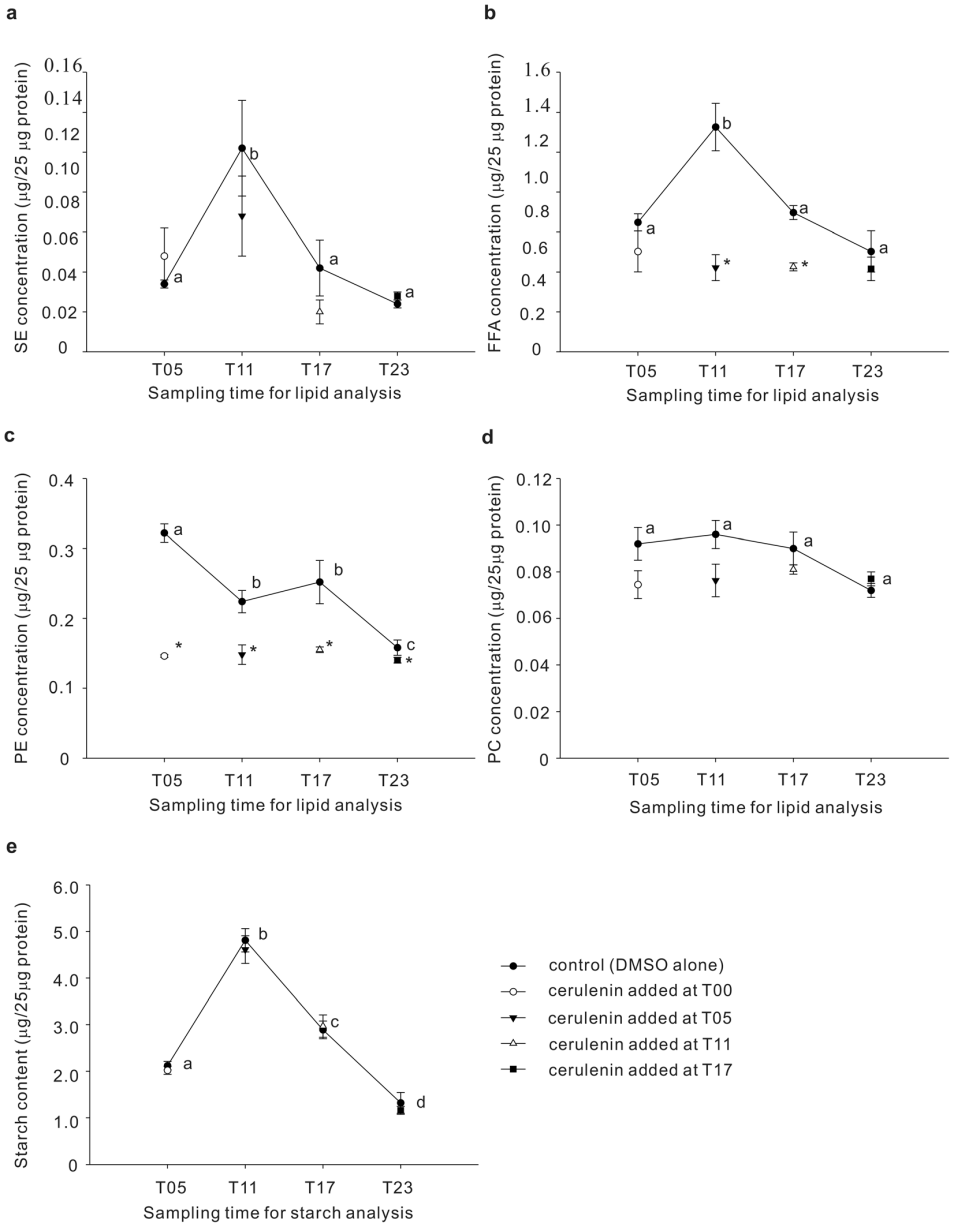
Comparison of lipid and starch content in control and cerulenin-treated *Symbiodinium* at different cell cycle stages. (a) SE; (b) FFA; (c) PE; (d) PC; (e) starch. Values were expressed as mean ± SEM. Letters (a–c and a'–c') denote the statistical significance of different time points according to one-way ANOVA followed by Duncan's multiple-range tests (*p*<0.05). * denotes the statistical significance of cerulenin treatment comparing to control at the same time point according student's t-test (*p*<0.05).

The lipid contents of *Symbiodinium* populations 5 or 6 h after cerulenin was added at T00, T05, T11 and T17 (i.e. T05, T11, T17 and T23, respectively) were analyzed. First, as shown in Fig. 5, there were dynamic concentration changes of SE, FFA, and PE, but not PC, in untreated *Symbiodinium* population. Although 0.02% DMSO was used as carrier in untreated *Symbiodinium* (see the “Materials and methods” section), the lipid concentration changes were intrinsic nature of the microalgal population, but not artifacts induced by DMSO (see Table S1). Both concentrations of SE (Fig. 5a) and FFA (Fig. 5b) increased significantly to the maximum level at T11 (0.108±0.021 and 1.126±0.138 μg/25 μg protein, respectively) and then decreased during T17 and T23 (SE: F = 4.460, *p* = 0.025; FFA: F = 9.400, *p* = 0.002). The concentration of PE gradually decreased over the 12L:12D cycle and reached the minimum level (0.158±0.015 μg/25 μg protein) at T23 (Fig. 5c; F = 10.765, *p* = 0.001). On the other hand, the concentration of PC remained unchanged throughout the 12L:12D cycle (Fig. 5d; F = 3.527, *p* = 0.052).

The SE concentration was not significantly changed after cerulenin treatment at T05, T11, T17, or T23 (Fig. 5a; T5: t = 1.938, *p* = 0.125; T11: t = 1.161, *p* = 0.290; T17: t = 1.239, *p* = 0.270; T23: t = 0.953, *p* = 0.378). Nevertheless, FFA concentrations at T11 and T17 significantly decreased (i.e. 0.422±0.065 and 0.426±0.020 μg/25 μg protein, respectively) when cerulenin was added at T05 and T11 for 6 h, compared to the control treatment (Fig. 5b) (T11: t = 4.481, *p* = 0.007; T17: t = 5.431, *p* = 0.003). Moreover, cerulenin treatment significantly decreased PE concentrations at T05 (0.146±0.002 μg/25 μg protein), T11 (0.148±0.014 μg/25 μg protein), T17 (0.155±0.004 μg/25 μg protein), and T23 (0.140±0.004 μg/25 μg protein) (Fig. 5c; t = 11.234, *p*<0.001; t = 3.195, *p* = 0.024; t = 2.594, *p* = 0.049; t = 6.631, and *p*<0.001, respectively). However, PC concentrations remained almost unchanged relative to the controls after treatments at T05, T11, T17, or T23 (Fig. 5d; t = 2.624, *p* = 0.059; t = 2.449, *p* = 0.058; t = 0.955, *p* = 0.3842; t = 1.166, *p* = 0.288, respectively). The result demonstrated that syntheses of specific lipid species, i.e. PE and FFA, were inhibited by cerulenin at different cell phases over the cell cycle.

### Effect of cerulenin on the starch content of cells of different phases of the cell cycle

The starch concentration of untreated *Symbiodinium* increased to the maximum level at T11 and then decreased to the basal level at T23 (Fig. 5e; F = 110.46, *p*<0.001). Starch concentrations also remained unchanged when *Symbiodinium* were treated with cerulenin at T05, T11, T17, and T23 for 6 h (t = 1.068, *p* = 0.310; t = 1.068, *p* = 0.311; t = 0.311, *p* = 0.762; t = 1.000, *p* = 0.341, respectively).

### Effects of cerulenin treatment on ingestion, distribution, and population density of *Symbiodinium* in *A. pulchella*

**Figure 6:**
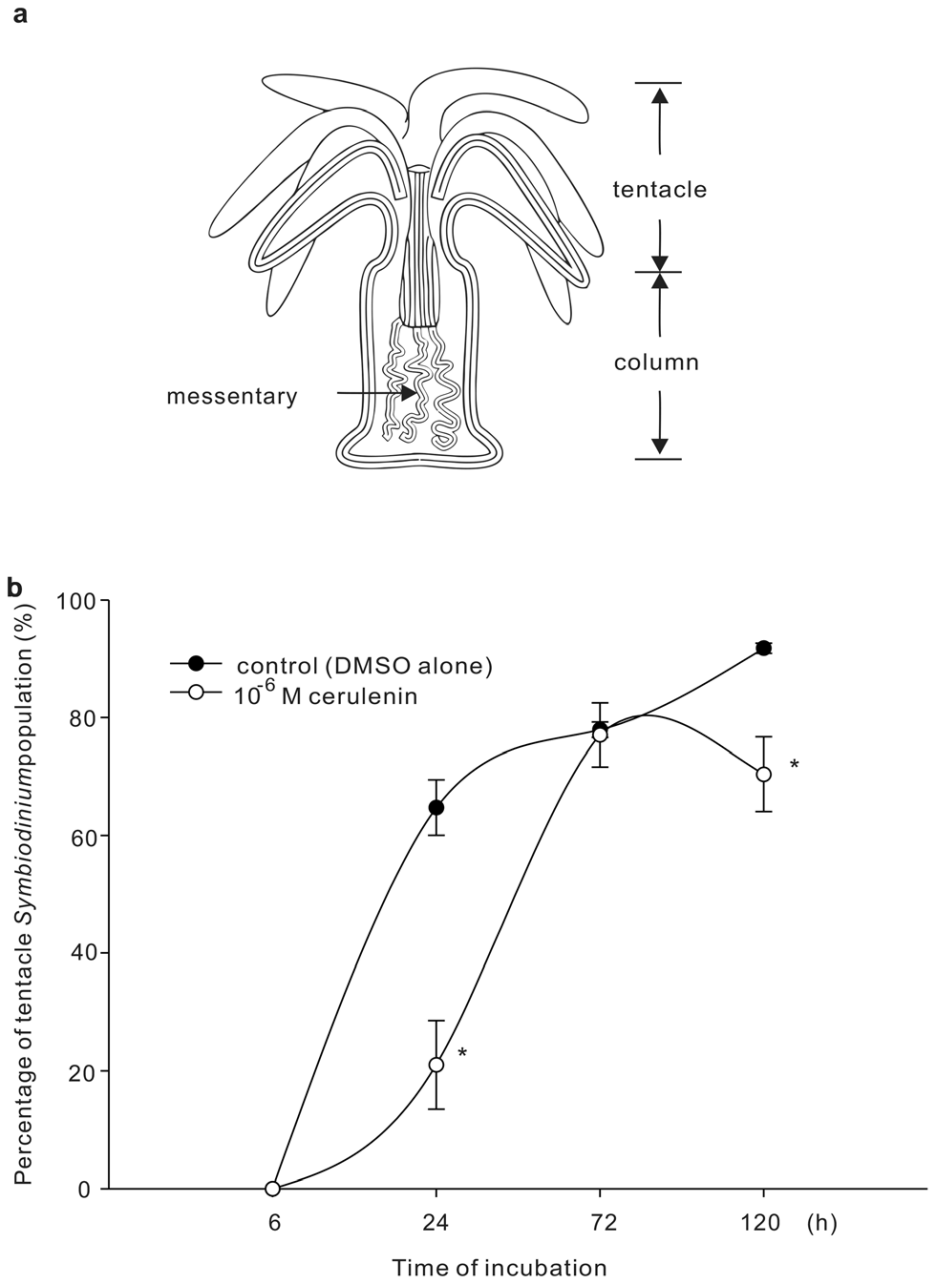
Effect of cerulenin on *Symbiodinium* distribution inside the host. (a) The schematic demonstration of dynamic *Symbiodinium* distribution from “column” to “tentacle” fractions during the infection process. (b) The effect of cerulenin treatment on the dynamic change of *Symbiodinium* population in tentacle fraction were examined. Percentage of tentacle fraction was calculated by dividing *Symbiodinium* number in tentacles with total number (i.e. numbers in tentacle plus column fractions, see also Table 2). All data are presented as mean ± SEM, *N* = 31. * denote the statistical significance of cerulenin treatment comparing to control at the same time point according Mann-Whitney Rank Sum Test (p<0.05).

As shown in Fig. 4, cerulenin treatments (10^−6^ M) at both T0 and T05 were able to arrest cells at G_1_. Furthermore, these arrested G_1_ cells contained significantly lower concentrations of both FFA and PE over the duration of the 12L:12D cycle (Fig. 5b–c). In order to elucidate how changes in lipid concentrations could affect the symbiotic association between *Symbiodinium* and anemones, *Symbiodinium* at T11, resulting from 11 h treatments of 0 (control) and 10^−6^ M cerulenin were collected to infect bleached anemones for 1 h in FSW. The number of ingested *Symbiodinium* and their distribution inside the host animal were then examined after 6, 24, 72, and 120 h after infection. Once the control *Symbiodinium* were ingested by the anemones, they first aggregated in the mesenteries of the host body column, and then later appeared in the tentacles (Fig. 6a). The cerulenin-treated *Symbiodinium* not only exhibited reduced uptake but also an abnormal distribution inside the anemone host, in comparison with control *Symbiodinium* (see Table 2 and Fig. 6b). First, the initial uptake of control *Symbiodinium* after 6 h of incubation was significantly higher than that of cerulenin-treated *Symbiodiniu*m (19±3 *vs.* 3±1 per anemone; p<0.001, see Table 2). During the following incubation, number of control *Symbiodinium* gradually increased and reached 1072±64 per anemone at 120 h, indicating rapid proliferation of the ingested microalgae inside the host. On the other hand, number of cerulenin-treated *Symbiodinium* proliferated slowly and only reached 19±4 per anemone after 120 h incubation. Secondly, there was a significant retardation of *Symbiodinium* population translocation from the mesenteries (i.e. the “column” fraction) toward the tentacle (Fig. 6b). After the first 6 h of incubation, all *Symbiodinium* distributed in the “column” fraction and no *Symbiodinium* could be identified in tentacle of the host (Fig. 6b; also see Table 2). After 24 h of incubation, 64.7±4.7% of the control *Symbiodinium* population redistributed from the mesenteries to the tentacles. On the other hand, most cerulenin-treated *Symbiodinium* still remained in the mesenteries, and only 21.0±6.2% of them had redistributed to the tentacles. The percentage of the cerulenin-treated *Symbiodinium* in the tentacles gradually increased to 77.1±5.6% after 72 h incubation, reaching a similar percentage as the controls.

**Table 2:** Progressional number changes of cerulenin-treated *Symbiodinium* per *A. pulchella* during the feeding process.a,b

Time distribution treatment	6 h			24 h			72 h			120 h		
	total	column	tentacle	total	column	tentacle	total	column	tentacle	total	column	tentacle
control (DMSO alone)	19±3	19±3	0±0	101±10	42±8	58±6	341±30.2	79±10	262±22	1072±64	81±7	991±64
10^−6^ M cerulenin	3±1*	3±1*	0±0	14±4*	12±4*	1±1*	24±5*	9±3*	15±4*	19±4*	5±2*	14±3*
t value	4.91	4.91	0	7.90	3.24	9.37	9.88	6.55	10.51	16.05	10.22	15.19
*p* value	<0.001	0.002	1.00	<0.001	0.002	<0.001	<0.001	<0.001	<0.001	<0.001	<0.001	<0.001

a The replication of each group at each time point was 31 (N = 31).

b Data represented as mean ± SEM.

* denotes the statistical significance of cerulenin treatment comparing to control at the same time point according student's t-test (*p*<0.05).

## Discussion

The cell cycle of free-living clade B *Symbiodinium* has been shown to be entrained by a 12L/12D photoperiod [16]. The light irradiation initiates the cell propagation from G_1_ to S, while dark treatment drives cells to progress toward G_2_/M and then cytokinesis. Furthermore, the cell motility is dynamic and increased during the first 7–8 h of light irradiation (G_1_ phase) relative to darkness [16]. In the heterotropic dinoflagellate, *Crypthecodinium cohnii*, cellular lipid profile varies with their cell cycle [4]. The present study shows that abnormal lipid synthesis not only affect the cell cycle of *Symbiodinium*, but also their ingestion and eventual symbiotic association with the sea anemone host.

Although starch content exhibited a diurnal pattern that coincided with cell cycle progression, starch content was not altered by cerulenin (Fig. 5e). The fact that cerulenin blocks cell cycle progression without altering starch content implies that newly synthesized lipids are more critical than starch during the cell cycle progression, especially at the G_1_/S and G_2_/M transitions. Four major lipids, including FFA, PE, PC and SE, were identified in *Symbiodinium*, with dynamic expression during the cell cycle. Among these lipids, cerulenin significantly inhibited synthesis of PE and FFA. *Symbiodinium* exhibited the highest PE content after being light-irradiated for 5 h, and levels of this lipid species then decreased over the course of the dark period (Fig. 5c). Similar concentration change of PE over the diel cycle was also observed during the vegetative development of *Chlamydomonas reinhardi* [27]. Such diel changes in PE production may be a consequence of the need for differing degrees of cellular membrane synthesis at different cell stages. In addition, local exposure of PE on the yeast plasma membrane involved in the polarized organization of the actin cytoskeleton and membrane curvature changes at the bud cortex of late mitotic cell, indicating the involvement of PE in cytokinesis [28]. As a consequence, inhibition of PE synthesis by cerulenin alters the cell cycle, as observed in the present study (Figs. 3–4).

Besides the PE synthesis, the biosynthesis of FFA turns out to be another critical regulation for cell cycle of *Symbiodinium*. Cerulenin deactivates three major types of fatty acid synthases [29]. Inhibition of FFA by cerulenin treatment is concurrent with G_1_ arrest or a transition delay from S to G_2_/M and G_2_/M to G_1_, depending on the time of cerulenin application (Figs. 3, 4, 5). Furthermore, in addition to the fatty acid synthases, the synthesis of FFA could be regulated by the acetyl-CoA carboxylase whose activity is protein kinase A (i.e. cAMP dependent protein kinase or PKA)-dependent [30]. As a consequence, the decrease of FFA synthesis by inhibiting PKA could alter the cell cycle. This was confirmed by a previous study showing that the inhibition of adenylyl cyclase (AC) resulted in the cell cycle arrest of clade B *Symbiodinium* at the G_1_/S transition [16].

The present study has also attempted to examine how alteration of lipid synthesis might affect *Symbiodinium* ingestion and symbiotic distribution in the host *A. pulchella*. After the initial ingestion, the *Symbiodinium*-gastroderm recognition in tentacles of the host is the next important task to establish highly specific mutualistic associations [31]. Although the cellular mechanisms underlying the ingestion/recognition between free-living *Symbiodinium* and cnidarian hosts remain unclear, the decreased ingestion of *Symbiodinum* induced by cerulenin treatment (see Table 2) was not due to different cellular motility upon the infection (see Figure S1). Wood-Charlson and colleagues have demonstrated that the glycan-lectin interaction may play a critical role during the recognition between the *Symbiodinium* and *Fungia scutaria* larvae [32]. The inhibition of *Symbiodinium* surface glycans, such as α-mannose/ α-glucose and α-galactose, by lectins greatly lowered their ingestions into the larvae [32]. Moreover, by binding surface galactose and glucosamine residues of *Symbiodinium* with a lectin analogue concanavalin A, the ingestion rate of *Symbiodinium* by the *Aiptasia pulchella* also significantly reduced [33]. These observations have collectively suggested that glycan ligands, such as α-mannose/ α-glucose and α-galactose, locating on the surface of *Symbiodinium* may play a role in the cell-cell recognition during the initial contact during the onset of symbiosis. Although the nature of *Symbidinium* surface glycans remains unclear, in the rhizobium–legume symbiosis, bacteria surface glycans (e.g. exo- and lipo-polysaccharides) of the soil bacteria (*Rhizobium meliloti* ) involving in symbiotic interaction with legumes were shown to anchore to the bacteria membranes via phospholipids [34]. The anchorage of these glycans required the FFA synthesis catalyzed by a specific type of fatty acid synthase [35]. As a consequence, the dynamic of PE and FFA components on *Symbiodinium* surface may be major regulators for ingestion and initial recognition during the onset of symbiosis.

## Supporting Information

Figure S1 **Effect of cerulenin (10^−6^ M) on** ***Symbiodinium*** **motility.** Cells were treated with or without cerulenin (10^−6^ M) at T00 (see Fig. 1). The percentage of motile cells was counted at different time using an epifluorescence microscope (Axiovision, Zeiss, Germany). Values were expressed as mean ± SEM. *N* = 5.
(EPS)

Table S1 (DOC)
