# Supplementary material for: Fatty Acid and Phospholipid Syntheses Are Prerequisites for the Cell Cycle of Symbiodinium and Their Endosymbiosis within Sea Anemones
Source: PLoS One. 2013 Aug 29;8(8):e72486. doi: 10.1371/journal.pone.0072486 (PMC3756969; doi:10.1371/journal.pone.0072486)
Supplement: Table S1 — (DOC) [file pone.0072486.s002.doc]

**Supplementary Table 1. Effect of DMSO on the lipid contents of cultured *Symbiodinium* sp.^abc^**

| Lipid class | SE content  (μg/25μg protein) | | FFA content  (μg/25μg protein) | | PE content  (μg/25μg protein) | | PC content  (μg/25μg protein) | |
| --- | --- | --- | --- | --- | --- | --- | --- | --- |
| time | control | 0.02%DMSO | control | 0.02%DMSO | control | 0.02%DMSO | control | 0.02%DMSO |
| T05 | 0.067±0.005 | 0.067±0.001 | 0.653±0.052 | 0.648±0.054 | 0.322±0.016 | 0.322±0.016 | 0.087±0.003 | 0.092±0.006 |
|  | t=0.08, *p*=0.94 | | t=0.06, *p*=0.95 | | t=0.85, *p*=0.44 | | t=0.96, *p*=0.39 | |
| T11 | 0.095±0.012 | 0.108±0.021 | 0.964±0.062 | 1.126±0.138 | 0.257±0.007 | 0.224±0.018 | 0.087±0.006 | 0.096±0.007 |
|  | t=0.54, *p*=0.61 | | t=1.07, *p*=0.33 | | t=1.67, *p*=0.16 | | t=1.36, *p*=0.23 | |
| T17 | 0.067±0.006 | 0.071±0.009 | 0.622±0.081 | 0.679±0.041 | 0.247±0.006 | 0.221±0.010 | 0.088±0.003 | 0.089±0.008 |
|  | t=0.39, *p*=0.71 | | t=0.08, *p*=0.94 | | t=2.10, *p*=0.10 | | t=0.16, *p*=0.88 | |
| T23 | 0.062±0.004 | 0.062±0.002 | 0.666±0.111 | 0.503±0.120 | 0.156±0.001 | 0.158±0.015 | 0.082±0.006 | 0.078±0.003 |
|  | t=0.02, *p*=0.98 | | t=1.05, *p*=0.34 | | t=0.75, *p*=0.49 | | t=1.52, *p*=0.19 | |

^a^The replication of each group at each time point was 3 (N=3).

^b^Data represented as mean ± SEM.

^c^The statistical significance comparing the treatments without (control) or with 0.02% DMSO at the same time point were analyzed according to Student’s t-test (p <0.05).
